# A decellularized human corneal scaffold for anterior corneal surface reconstruction

**DOI:** 10.1038/s41598-021-82678-3

**Published:** 2021-02-04

**Authors:** Naresh Polisetti, Anke Schmid, Ursula Schlötzer-Schrehardt, Philip Maier, Stefan J. Lang, Thorsten Steinberg, Günther Schlunck, Thomas Reinhard

**Affiliations:** 1 Eye Center, Medical Center - Faculty of Medicine, University of Freiburg, Killianstrasse 5, 79106 Freiburg, Germany; 2 Department of Ophthalmology, University Hospital Erlangen, Friedrich-Alexander-University of Erlangen-Nürnberg, Schwabachanlage 6, 91054 Erlangen, Germany; 3 Department of Operative Dentistry and Periodontology, Division of Oral Biotechnology, University of Freiburg, Hugstetter Strasse 55, 79106 Freiburg, Germany

**Keywords:** Tissue engineering, Biomaterials - cells

## Abstract

Allogenic transplants of the cornea are prone to rejection, especially in repetitive transplantation and in scarred or highly vascularized recipient sites. Patients with these ailments would particularly benefit from the possibility to use non-immunogenic decellularized tissue scaffolds for transplantation, which may be repopulated by host cells in situ or in vitro. So, the aim of this study was to develop a fast and efficient decellularization method for creating a human corneal extracellular matrix scaffold suitable for repopulation with human cells from the corneal limbus. To decellularize human donor corneas, sodium deoxycholate, deoxyribonuclease I, and dextran were assessed to remove cells and nuclei and to control tissue swelling, respectively. We evaluated the decellularization effects on the ultrastructure, optical, mechanical, and biological properties of the human cornea. Scaffold recellularization was studied using primary human limbal epithelial cells, stromal cells, and melanocytes in vitro and a lamellar transplantation approach ex vivo. Our data strongly suggest that this approach allowed the effective removal of cellular and nuclear material in a very short period of time while preserving extracellular matrix proteins, glycosaminoglycans, tissue structure, and optical transmission properties. In vitro recellularization demonstrated good biocompatibility of the decellularized human cornea and ex vivo transplantation revealed complete epithelialization and stromal repopulation from the host tissue. Thus, the generated decellularized human corneal scaffold could be a promising biological material for anterior corneal reconstruction in the treatment of corneal defects.

## Introduction

Allogenic corneal transplantation is a highly successful technique to restore vision in patients with corneal diseases or after ocular trauma^1^. In eyes with moderate damage, the ocular immune privilege and the lack of corneal blood vessels decrease the risk for immunological transplant rejection. This is different in eyes with severe corneal damage, e.g. in patients suffering from alkaline burns or corneal stromal ulceration due to inflammation or infection, where allogenic transplants placed in an inflamed recipient bed are prone to transplant rejection^2,3^. These patients in particular would benefit from the possibility to use non-immunogenic decellularized tissue scaffolds which may be repopulated by host cells in situ. A large number of studies used porcine tissue for decellularization^4,5^. However, its clinical implementation may be hampered by issues of xenotransplantation such as immunocompatibility and the possible presence of porcine viruses. Human donor corneas unsuitable for transplantation due to a low endothelial cell count have therefore been advocated as an excellent source of the tissue^6,7^. With the broad use of Descemet membrane endothelial keratoplasty (DMEK), where only a 20–30 µm thick tissue membrane and the endothelial cell layer are being transplanted, a large number of corneal tissue remnants remain after DMEK transplant preparation and could also be used for scaffold preparation to treat corneal ulcers and superficial defects.

Several methods have been described to decellularize corneal tissue using physical or chemical means^8,9^. For decellularization, hypertonic sodium chloride (NaCl), sodium dodecyl sulfate (SDS) or non-ionic detergents such as Triton X-100 have been used alone or in combination with nucleases to eradicate cells while preserving protein content and structure of the extracellular matrix (ECM) scaffold to varying degrees^6,7^. Drawbacks may be that SDS denatures proteins and a decrease in scaffold transparency was reported after treatment with SDS or hypertonic salt^7^. Moreover, protocols using SDS or hypertonic salt with nucleases are very time-consuming (~ 5–8 days)^6,7,10^ and failure to fully elute SDS can have cytotoxic effects^6,7^. Sodium deoxycholate (SD), a naturally occurring bile salt metabolite, has been used to decellularize various organs or tissues such as liver^11^, peripheral nerves^12^, ovary^13^, uterus^14^, conchal cartilage^15^, trachea^16^, lungs and heart valves^17^. The combination of SD/ deoxyribonuclease I (DNAse) was also used to generate decellularized human corneal limbus, however, a long elution time for removal of cellular components was a drawback of the protocol suggested^18^. Moreover, there are no reports on a combination of SD and DNAse as a decellularizing agent for human corneal tissue. Synthetic SD is being applied clinically as an injectable dissolvent of subcutaneous body fat^19^ and was FDA-approved for removal of submental fat, which may illustrate its biocompatibility. The cornea is endowed with high glycosaminoglycan content and it has been shown that tissue swelling during processing can cause structural damage, which was prevented by adding dextran to the solutions used on porcine corneal tissue^20^. The role of dextran and tissue swelling during human corneal decellularization has not specifically been addressed in earlier protocols^6,7^. Moreover, published decellularization protocols often lack an extensive characterization of ECM integrity and preservation of BM components^6,7^, which are essential to maintain tissue homeostasis and ensure cell adhesion, growth, long term survival and function of corneal epithelium and epithelial progenitor cell phenotype^8,21,22^. SDS and triton-x-100 were shown to be cytotoxic and limit biocompatibility^7,23^.

In light of these data, it was our goal to devise a further improved protocol for decellularization of human corneal tissue remnants after removal of transplant material for DMEK (without Descemet membrane, labeled DM^−^) using agents in clinical use and controlling for tissue swelling. We also wanted to clarify whether corneal tissue unsuitable for transplantation due to a low endothelial cell count (with Descemet membrane, labeled DM^+^), would be amenable to the same protocol with similar preservation of tissue integrity. We evaluated decellularization effects on the basement membrane (BM) components and physical characteristics such as transparency and mechanical properties of the decellularized human cornea (DHC). Furthermore, we evaluated the biocompatibility of generated DHC scaffolds by repopulation with human corneal cells from the limbus.

## Results

### Decellularization of human corneas

**Figure 1:**
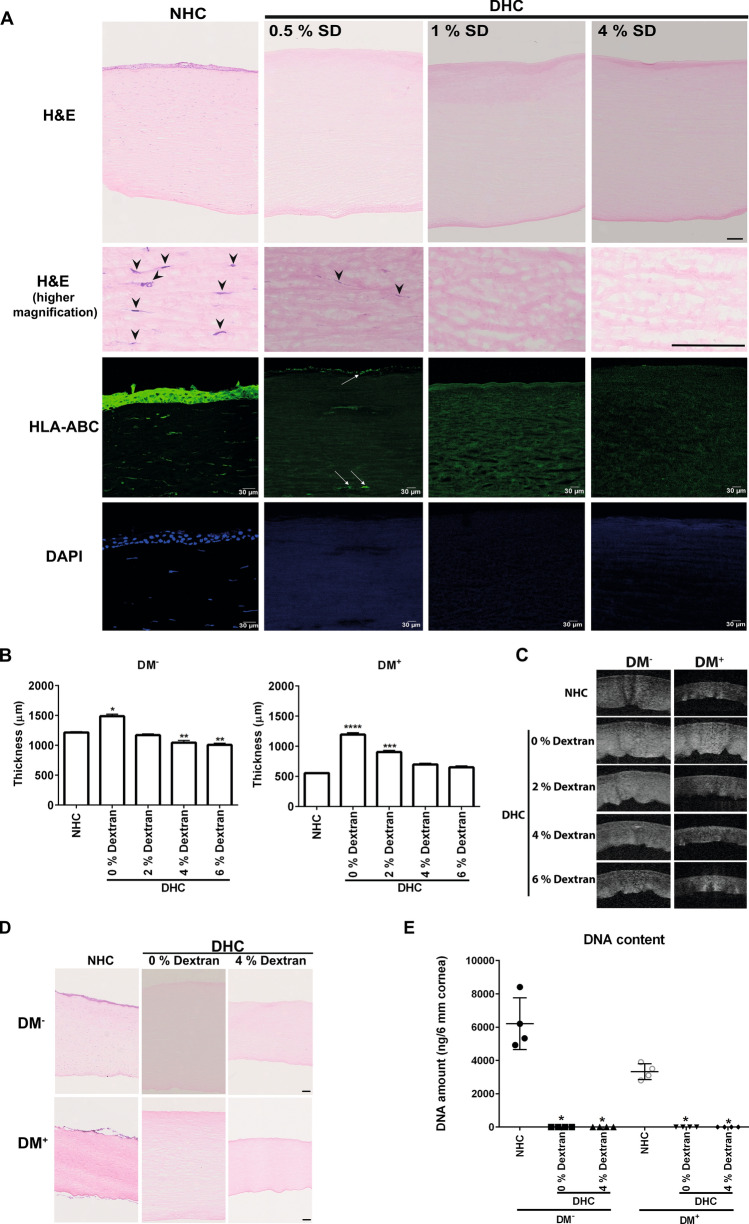
Decellularization efficiency and role of dextran: (**A**) Histological evaluation of human cornea decellularized by various concentrations of sodium deoxycholate (SD) with deoxyribonuclease I in comparison to the normal human cornea (NHC) using hematoxylin and eosin (H&E) staining. The higher magnification images of H&E staining showing the stromal cells in NHC (arrowheads) and cellular debris in 0.5% SD ( arrowheads). Scale bar = 100 µm. HLA-ABC staining (green) of NHC and DHC showing cellular expression of HLA in NHC and 0.5% SD treated DHC (white arrows); no HLA expression in 1% and 4% SD treated DHC cornea. DAPI staining reveals nuclei in NHC without remaining nuclei/nuclear debris in DHC. (**B**) The role of dextran during decellularization in both DM^−^ and DM^+^ cornea in comparison to respective controls. Graphs represent the thickness of DHC in various dextran conditions compared to NHC. Data are expressed as means ± S.E.M. (n = 5). * *p* < 0.05; ** *p* < 0.01; *** *p* < 0.001; **** *p* < 0.0001; (Wilcoxon signed-rank test). (**C**) AS-OCT images of DM^−^ and DM^+^ cornea in varying concentrations of dextran with respective controls. (**D**) Histological evaluation by staining with H&E on NHC and DHC showing no cell remnants in DHC but varying corneal thickness (in presence or absence of dextran) of both DM^−^ and DM^+^ cornea. Scale bar = 100 µm E) Confirmation of decellularization by quantification of residual DNA. The graph represents the quantity of DNA in both DM^−^ and DM^+^ cornea before and after decellularization (0 or 4% dextran). Data are expressed as means ± S.E.M. (n = 4). * *p* < 0.05; Mann–Whitney U test. *DAPI* 4′,6‐diamidino‐2‐phenylindole, *DM*^*−*^ cornea with the absence of Descemet membrane, *DM*^+^ cornea with the presence of Descemet membrane, *AS-OCT* anterior segment optical coherence tomography, *HLA* human leukocyte antigen.

Human DM^−^ corneas decellularized with different concentrations of SD were initially screened by hematoxylin and eosin (H&E) staining, immunofluorescent staining for human leukocyte antigen (HLA)-ABC was used to assess the presence of residual cellular/membranous material, and nuclear staining with 4′,6-diamidino-2-phenylindole (DAPI) (Fig. 1A). H&E staining showed distinctly visible corneal epithelial and stromal cells (arrowheads indicating stromal cells at higher magnification) in normal human cornea (NHC) (Fig. 1A). In DHC treated with 4% SD or 1% SD, no epithelial and stromal cells were observed and HLA-ABC, as well as deoxyribonucleic acid (DNA) (DAPI), was also not detected (Fig. 1A). The use of 0.5% SD did not result in complete removal of cellular debris as apparent in H&E (Fig. 1A, higher magnification, arrowheads) and HLA-ABC staining’s (Fig. 1A, arrows). These results were reproduced in DM^+^ corneas (data not shown). Thus, a concentration of 1% SD was used for decellularization in all subsequent experiments. We did observe an increased thickness of DHC as compared to the respective NHC controls. Final swelling states of DHC in the DM^−^ and DM^+^ groups were also different depending on the swelling state at the outset of the decellularization procedure (Table 1).

**Table 1:** Corneal thickness measurements by anterior segment optical coherence tomography.

Corneal tissue	Treatment	Dextran during treatment (%)	Thickness (µm) after treatment (mean ± S.D.)	Thickness (µm) after additional 24 h in 6% dextran (mean ± S.D.)
DM^−^	NHC	0	1243.4 ± 108.3	
	Dextran Control	6	702.6 ± 57.4 (24 h)	548.0 ± 19.2 (48 h)
	DHC	0	1487.6 ± 103.2	686.3 ± 35.0
		2	1169.9 ± 71.7	609.0 ± 11.9
		4	1043.7 ± 112.7	536.6 ± 24.7
		6	1005.8 ± 96.2	540.1 ± 30.5
DM^+^	NHC	6	554.1 ± 55.4	
	Dextran-free Control	0	711.2 ± 86.6 (24 h)	528.0 ± 31.9
	DHC	0	1192.1 ± 125.2	477.0 ± 37.5
		2	902.2 ± 95.4	581.3 ± 36.2
		4	679.2 ± 59.7	536.3 ± 9.2
		6	649.4 ± 76.9	593.6 ± 10.5

### Effects of dextran during decellularization

In DM^−^ corneas, the average thickness of control NHC was 1243.4 ± 108.3 µm, whereas DHC processed in the absence of dextran (1487.6 ± 103.2 µm, *p* = 0.03) were significantly thicker (Fig. 1B). Decellularization in 2% dextran yielded thinner scaffolds (1169.9 ± 71.7 µm), in 4% dextran (1043.7 ± 112.7 µm, *p* = 0.007) and 6% dextran (1005.8 ± 96.2 µm, *p* = 007) the reduction in corneal thickness was significant when compared to (NHC) (Fig. 1B). As dextran control, the NHC were incubated in a medium containing 6% dextran with an incubation time equivalent to the decellularization process (~ 24 h) resulted in a significant decrease in thickness (702.6 ± 57.4 µm, *p* = 0.0001) (Table 1).

In DM^+^ cornea, the average thickness of NHC was 554.1 ± 55.4 µm, whereas DHC were swollen in the absence of dextran (1192.1 ± 125.2 µm, *p* = 0.0001) and less so in 2% dextran (902.2 ± 95.4 µm, *p* = 0.003, Fig. 1B). The mean thickness of the scaffolds decellularized using 4% dextran (679.2 ± 59.7 µm) and 6% dextran (649.4 ± 76.9 µm) was best comparable to NHC (Fig. 1B). As dextran-free control, the NHC were incubated in a medium containing 0% dextran with an incubation time equivalent to the decellularization process (~ 24 h) resulted in a significant increase in thickness (711.2 ± 86.6 µm, *p* = 0.0001) (Table 1).

The corresponding anterior segment optical coherence tomography (AS-OCT) images also reflected the same (Fig. 1C). When corneas (both DM^−^ and DM^+^) were kept at 6% dextran for 24 h after decellularization, corneal thickness was comparable to NHC irrespective of the dextran concentration used in decellularization (Table 1). Based on these results, we performed all subsequent experiments using 1% SD with 4% dextran during the whole decellularization process, compared to 1% SD without dextran. Even though the 2% dextran was effective to reduce the thickness of DM^−^ corneas (1169.9 ± 71.7 µm) to match NHC (1243.4 ± 108.3 µm), we used 4% dextran for all further experiments as it was our goal to devise a protocol amenable to both DM^−^ and DM^+^ corneas.

To test whether the decellularization in the presence of 4% dextran is as efficient as in its absence, histological evaluations were performed in both DM^−^ and DM^+^ corneas. H&E staining showed a decrease of corneal thickness when dextran was present during decellularization, but no differences were noticed with respect to remnant cellular material. This was true in DM^−^ as well as DM^+^ samples (Fig. 1D). Spectroscopic analyses of DNA content showed significantly less residual DNA in DHC compared to NHC, irrespective of dextran used during decellularization. SD and DNAse treatment removed about 98.6 ± 1.2% of the DNA content in the presence of dextran and 98.4 ± 0.9% in its absence (Fig. 1E). We also noticed that the quality of DM^+^ corneas was poor compared to DM^−^ (loss of epithelial layers in (Fig. 1D), and showed lower DNA content (Fig. 1E) presumably due to long time incubation in dextran containing medium as mentioned earlier (Table 2)^24^.

**Table 2:** Organ cultured corneal sample details.

Corneal samples	Number	Donor age (years)*	Cultivation duration (days)*	Cultivation in dextran (days)*
Total	**183**	**70.2 ± 13.0 (24–93)**	**32.0 ± 9.5 (6–75)**	** 4.4 ± 6.5 (0–43)**
DM^−^	102	69.4 ± 10.3(42–87)	30.0 ± 6.4 (16–58)	1.4 ± 1.1 (0–3)
DM^+^	51	77.3 ± 11.4 (50–93)	35.3 ± 11.7 (6–69)	12.5 ± 8.4 (3–43)
After PK	30	61.3 ± 16.7 (24–86)	35.0 ± 14.6 (20–75)	2.3 ± 1.1 (1–4)

*All values are expressed as mean ± standard deviation (range).

### Scanning electron microscopy

**Figure 2:**
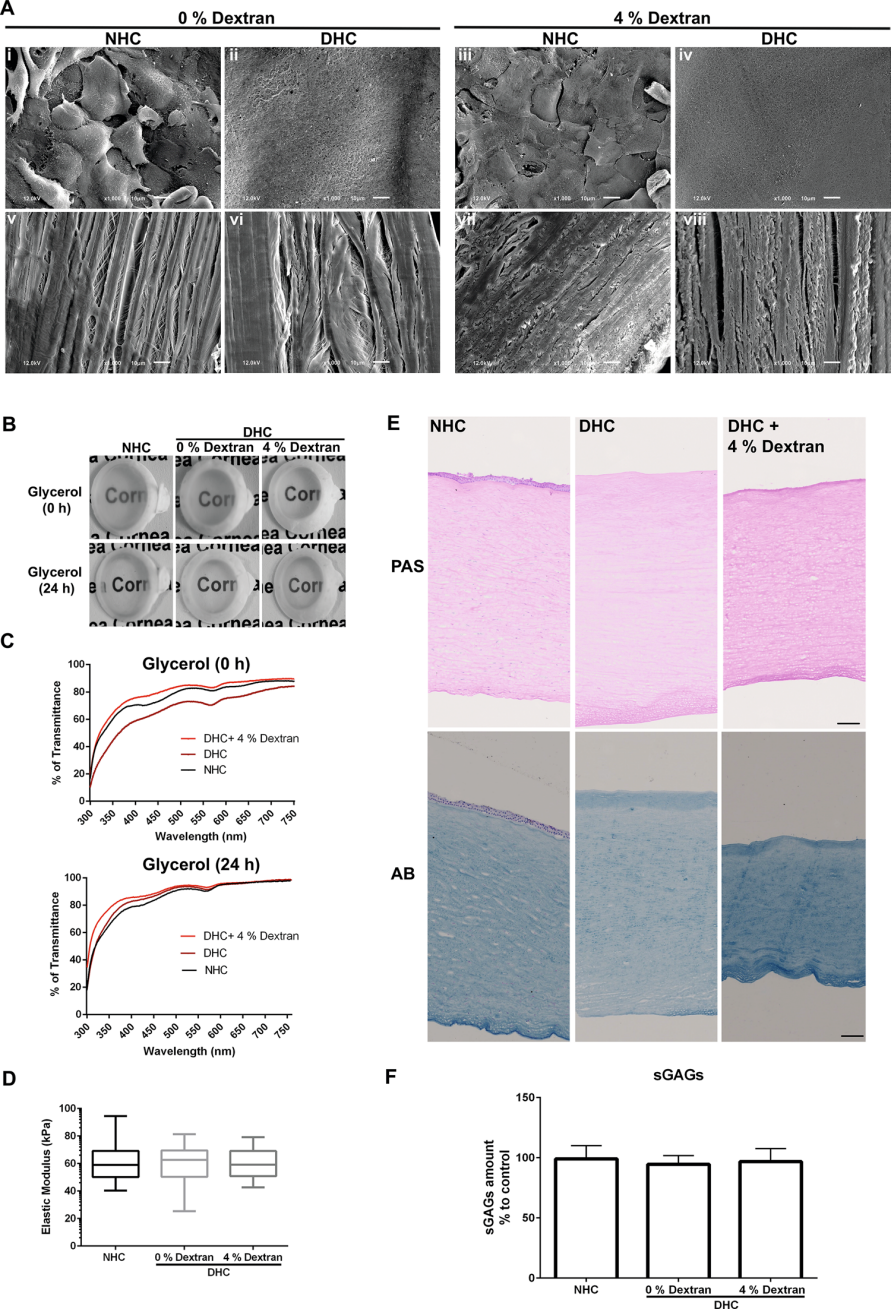
Physical and biochemical properties of the decellularized human cornea (DHC). (**A**) Scanning electron microscopy micrographs of the normal human cornea (NHC) and DHC in presence or absence of dextran; the anterior surface of the cornea (i–iv) showing cells on the NHC (I, iii) but no cells on the DHC (ii, iv) and cross-section of the cornea (v–viii) showing collagen bundles and distance between the bundles being larger without dextran (i, ii). Scale bar = 10 µm. (**B**) Macroscopic pictures of NHC & DHC (0, 4% dextran) placed on the word “cornea” in the background for a visual representation of the tissue transparency: DHC seems to be a bit cloudier without dextran than NHC and DHC with 4% dextran. (**C**) Quantitative analysis of light transmittance through the NHC and DHC in glycerol before and after incubation at visible wavelengths (n = 5). The graph represents the percentage of transmittance in NHC and DHC at different wavelengths. (**D**) Mechanical properties of DHC by indentation in comparison to NHC. Data are expressed as means ± S.E.M. (n = 4). (**E**) Histological evaluation of extracellular matrix content by periodic acid Schiff (PAS) and alcian blue (AB) on DHC (with 0 or 4% dextran) compared with NHC. Scale bar = 100 µm. (**F**) Sulfated glycosaminoglycans (sGAGs) content of DHC in the presence or absence of dextran in comparison to NHC. The graph represents a percentage (%) of sGAGs content in corneal samples and data are expressed as means ± S.E.M. (n = 5).

The effect of decellularization treatment on the integrity of the ECM structure was evaluated by scanning electron microscopy (SEM). As dextran control, the NHC were incubated in a medium containing 4% dextran with an incubation time equivalent to the decellularization process. Epithelial cells detectable in control corneas (Fig. 2A, i & iii) were absent in DHC (Fig. 2A, ii & iv), irrespective of dextran use. On cross-sections, no significant gross changes of the collagen fibers were noted in DHC except that the collagen bundles were slightly larger due to swelling and a slight increase in distance between bundles in absence of dextran (Fig. 2A, vi). When decellularized in the presence of 4% dextran, DHC showed a decrease in collagen bundles distance (Fig. 2A, viii) compared to NHC in dextran-free medium (Fig. 2A, v).

### Optical properties

Macroscopic tissue evaluation suggested that decellularization in the presence of dextran did not affect transparency or even slightly improved it, whereas decellularization in the absence of dextran led to some tissue clouding (Fig. 2B, 0 h). Twenty-four hours of incubation in glycerol resulted in the recovery of DHC transparency in the absence of dextran (Fig. 2B, 24 h). The spectroscopic analysis at 300–750 nm revealed similar transparency of NHC and DHC processed in 4% dextran (Fig. 2C). In the absence of dextran, there was a reduction in transparency by 10–15% (Fig. 2C, 0 h), which subsided after 24 h in glycerol (Fig. 2C, 24 h).

### Mechanical properties

Instrumented indentation measurements indicated no major differences in the elastic moduli of NHC (62.6 ± 15.9 kPa) and DHC irrespective of dextran (59.3 ± 13.6 kPa in 0% dextran; 59.2 ± 10.56 kPa in 4% dextran) used during decellularization (Fig. 2D).

### ECM components

In periodic acid schiff (PAS) staining, the intensity of the glycoproteins of DHC seemed markedly reduced in the absence of dextran as the thickness of the cornea also increased (Fig. 2E) whereas the intensity was increased in the DHC in the presence of dextran (Fig. 2E), compared to NHC as thickness also decreased (Fig. 2E).

Alcian Blue (AB) staining of NHC showed the presence of glycosaminoglycan (GAG) throughout the cornea. In DHC, GAG staining intensity was much weaker when treated in the absence of dextran (Fig. 2E), whereas in DHC treated in the presence of dextran GAG staining was more intense than in NHC (Fig. 2E). The levels of sulfated GAG (sGAG) measured with biochemical assays were unaffected by decellularization and no significant differences were observed among the samples (Fig. 2F).

The ECM of the corneal surface was evaluated in detail by immunostaining for various components including agrin, collagen types (Col III, IV, XVIII), fibronectin (FN), junctional adhesion molecule C (JAM-C), perlecan, tenascin C (TN-C), vitronectin (VN), and laminin (LN) chains (α3, α5, β2, β3, and γ2).

**Figure 3:**
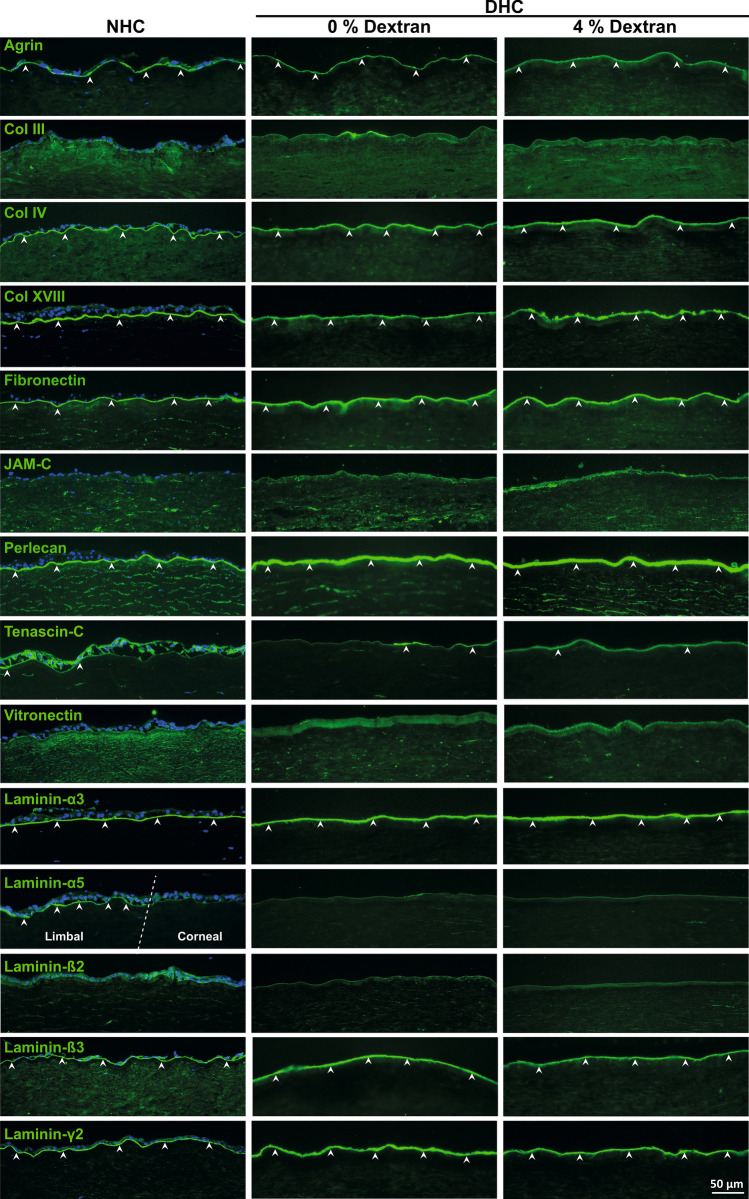
Extracellular matrix (ECM) composition of decellularized human cornea (DHC). Immunostaining analysis of various (ECM) and basement membrane (BM) molecules on the DHC (0 and 4% dextran) compared to the normal human cornea (NHC). The white dashed line marked the boundary between the limbal and corneal tissue in laminin (LN) α5 staining of NHC. Arrowheads indicating the expression of ECM and BM molecules. Nuclei counterstained with 4′,6‐diamidino‐2‐phenylindole (blue). *Col* Collagen, *JAM-C* junctional adhesion molecule C.

The major BM heparan sulfate proteoglycan, agrin, was expressed in BM with uniform and strong labeling intensity in all samples (Fig. 3, arrowheads). Expression of Coll IV and Coll XVIII molecules were clearly noted in BM of the cornea in both NHC and DHC samples (Fig. 3, arrowheads). No significant difference was noted for Col IV, whereas Coll XVIII expression was reduced in DHC (no dextran) and discontinuous in DHC (4% dextran) compared to NHC. The fibrillar Coll III was not observed in either NHC or DHC (Fig. 3).

FN was clearly detected in BM in all samples without any significant differences (arrowheads), whereas a weak expression of vitronectin was observed (Fig. 3). Strong expression of the other major BM heparan sulfate proteoglycan, perlecan, was noted similar to agrin (Fig. 3, arrowheads). TN-C was hardly expressed on the BM of the corneal surface of all samples, whereas JAM-C expression was not observed (Fig. 3). Using Laminin chain specific antibodies, the LN-α3, -ß3, -γ2 chains were found to be strongly expressed in the corneal BM of NHC and DHC with or without dextran (Fig. 3, arrowheads). No significant differences were observed in staining patterns among samples. The LN α5 chain and β2 chains were not detectable in corneal samples as their expression is known to be limited to the limbal region (Fig. 3)^25^.

### Recellularization

The characterization details of cultured limbal epithelial progenitor cells (LEPC), limbal mesenchymal stromal cells (LMSC), and limbal melanocytes (LM) were provided in the Supplementary Data S1.

### In vitro recellularization

**Figure 4:**
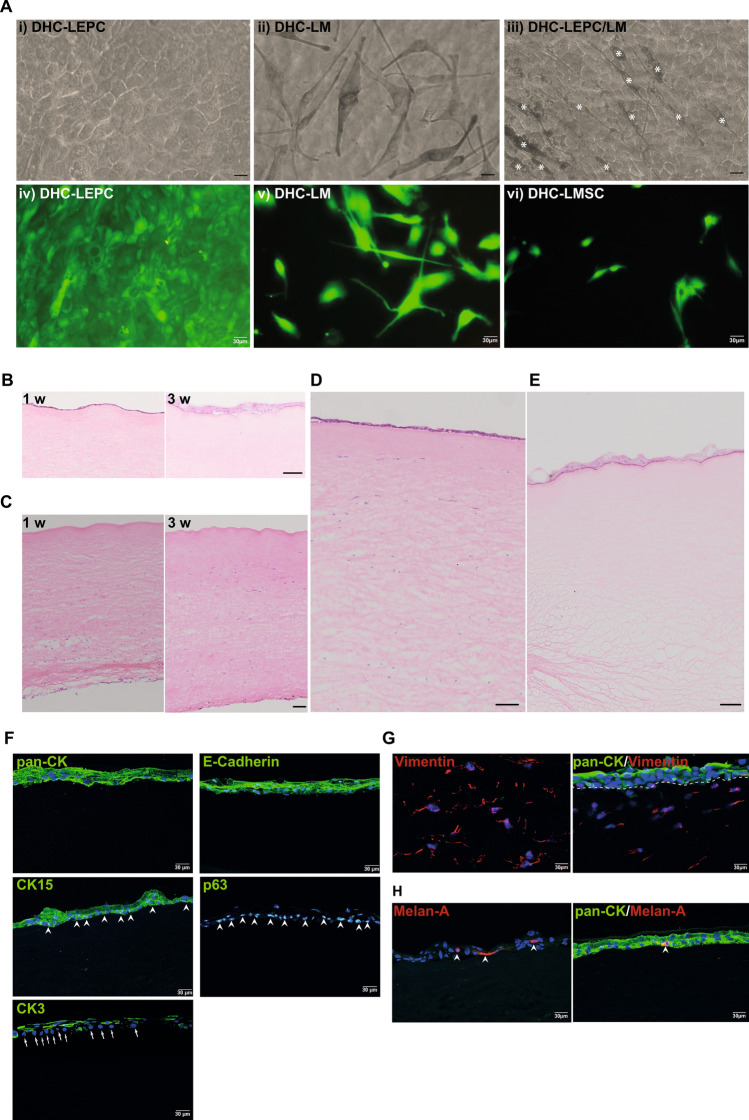
In vitro recellularization of the decellularized human cornea (DHC) (**A**) Phase contrast micrographs of LEPC (i); LM (ii); and LEPC/LM scaffolds showing intermingled melanocytes (iii, white asterisk) in the epithelial cell layer on DHC. Scale bar = 20 µm. Live/dead viability assay after 24 h of cultivation of LEPC, LMSC, and LM on DHC (iv–vi). (**B**) Light microscopic analysis of DHC-LEPC scaffolds showing cell monolayers after 1 week (1 w) and stratified epithelium (3 w) by hematoxylin and eosin staining (H&E) on the anterior surface. Scale bar—100 µm. (**C**) The injected stromal cells in DHC showing the stromal cells on the posterior side at 1-week culture and migrated to the anterior side after 3 weeks by H&E staining. Scale bar—100 µm (**D**) DHC-LEPC/LMSC scaffold after 3 weeks cultivation showing stratified epithelium and presence of stromal cells throughout the stroma by H&E staining (**E**) DHC-LEPC/LM scaffold showing stratified epithelium after 3 weeks of cultivation. Scale bar—100 µm (**F**) Immunofluorescence staining of DHL-LEPC scaffolds showing the expression of CK15 and p63 (green) at basal layers of epithelial cells (arrowheads); CK3 expression (green) in suprabasal cells but not in basal cells (arrows); E(epithelial)-cadherin and cytokeratin (pan-CK) expression (green) in all human limbal epithelial cells on DHC. (**G**) DHL-LEPC/LMSC scaffolds showing the vimentin expression on stromal cells and cytokeratin (pan-CK) expression (green) on epithelial layers. The dashed line separated the epithelium and stroma. (**H**) DHL-LEPC/LM scaffolds showing the melan-A (red) positive melanocytes (arrowheads) in the epithelial layers (pan-CK, green). *LEPC* limbal epithelial progenitor cells; *LMSC* limbal mesenchymal stromal cells, *LM* limbal melanocytes, *pan-CK* pan-cytokeratin, *CK15* cytokeratin 15, *CK3* cytokeratin 3, *DAPI* 4′,6‐diamidino‐2‐phenylindole.

The biocompatibility of the DHC to support LEPC, LMSC, and LM was evaluated. The seeded LEPC and LMs attached to the surface of decellularized corneas (Fig. 4A, i-iii) and remained viable after 48 h of cultivation as confirmed by live/ dead staining (Fig. 4A, iv&v). In DHC-LEPC/LM scaffolds, the melanocytes intermingled with epithelial cells (Fig. 4A, iii, white asterisk). The LMSCs injected into DHC spread and appeared healthy (Fig. 4A, vi). H&E staining showed a monolayer of LEPC on DHC after 1 week of cultivation (Fig. 4B, 1 w). After 3 weeks in culture stratification of the epithelium was successfully induced by lifting the tissues to the air–liquid interface and confirmed by H&E staining (Fig. 4B, 3 w). The injected LMSCs were spread over the posterior side of the DHC after 1 week (Fig. 4C, 1 w). After 3 weeks, the LMSCs appeared to migrate into the anterior part of the corneal stroma (Fig. 4C, 3 w). After 3 weeks of cultivation, the DHC-LEPC/LM scaffolds showed a stratified surface epithelium and stromal cells in the anterior part of the stroma as confirmed by H&E staining (Fig. 4D). The DHC-LEPC/LM scaffolds also showed a stratified epithelium after 3 weeks of cultivation (Fig. 4E).

Phenotypic evaluation of recellularized scaffolds by immunofluorescent staining confirmed pronounced pan-cytokeratin (pan-CK) and E(epithelial)-cadherin staining in all epithelial layers; CK15 and p63 staining confined to basal layers (Fig. 4F, arrowheads); corneal differentiation marker CK3 was expressed in superficial epithelial layers but not in basal cells (Fig. 4F, arrows indicating basal cells) in DHC-LEPC scaffolds . Vimentin-positive cells (red) were observed in the stromal region close to pan-CK-positive epithelial cells (green) of the DHC-LEPC/LMSC scaffolds (Fig. 4G). Melan-A positive cells (red) were interspersed in the epithelial layers (pan-CK^+^, green) of the DHC-LEPC/LM scaffolds (Fig. 4H, arrowhead). The results of recellularization by neighboring tissue are provided in Supplementary Data S2.

### Ex vivo transplantation

**Figure 5:**
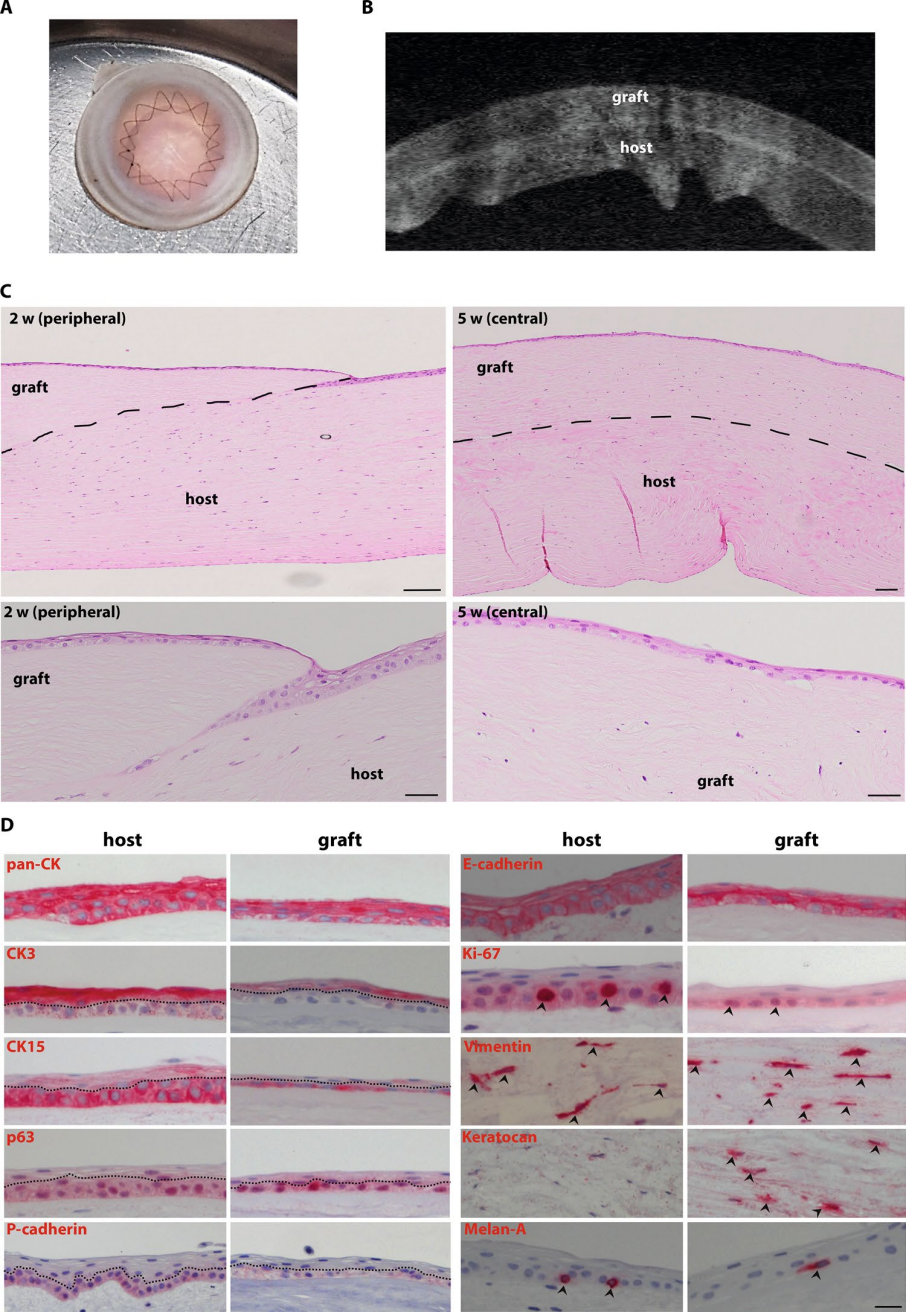
Ex Vivo transplantation of decellularized human cornea (DHC) (**A**) micrograph of DHC transplanted ex vivo in the normal human cornea (NHC) as anterior lamellar grafts with double running cross-stitch sutures. (**B**) AS-OCT micrograph showing the well-adapted graft (DHC) and host tissue (NHC) and no gap was observed in between. (**C**) After 2 weeks (2 w) of transplantation, histological analyses by hematoxylin and eosin (H&E) staining showed complete epithelialization of graft tissue with stratification and absence of stromal cells in graft tissue (2 w) (dashed line marking the boundary between the graft and host tissue). The stromal cells appeared in the graft region at 5 weeks samples confirmed by H&E staining (5 w). Scale bar—100 µm D) Immunohistochemical staining on paraffin sections showing the cytokeratin (pan-CK) expression on epithelial layers; CK3 expression on suprabasal cells; CK15, P(placental)-cadherin, p63 (dotted line separated the basal and superficial cells), and Ki-67 expression at basal layers of epithelial cells (arrowheads); E(epithelial)-cadherin expression in all epithelial layers; vimentin expression in the stromal region (arrowheads); and Melan-A expression (magenta) (arrowheads) on melanocytes at basal layers of epithelium, in both host and graft tissue. A keratocan expression was observed only in the graft tissue (arrowheads). *AS-OCT* anterior segment optical coherence tomography, *pan-CK* pan-cytokeratin, *CK3* cytokeratin 3, *CK15* cytokeratin 15.

The anterior part of a DHC was successfully sutured onto the posterior bed of a NHC (Fig. 5A) and both tissues were well adapted by sutures as confirmed by AS-OCT imaging (Fig. 5B). The surgical handling of the DHC tissue was comparable to NHC and no significant tissue damage occurred during suturing. After 2 weeks in culture, histological analyses showed complete epithelialization of the grafted tissue scaffold with stratification and the absence of cells in the stromal region of the graft (Fig. 5C, 2 w). Stromal cells were detectable in the graft 5 weeks after surgery (Fig. 5C, 5 w). Immunohistochemical analyses revealed the expression of the epithelial markers cytokeratin (pan-CK) and E-cadherin in the epithelial cells on the host (peripheral cornea/limbal region) and graft tissue (Fig. 5D). Expression of the LEPC markers, CK15, P(placental)-cadherin, and p63, was apparent in basal epithelial cells on both, the grafted scaffold and host tissue, whereas expression of the corneal differentiation marker CK3 was present only in the superficial epithelial cells of the graft and host tissue (Fig. 5D). Moreover, we also observed Ki-67 positive cells in the basal layer of the epithelium covering the DHC tissue (Fig. 5D, arrowheads). The migrated stromal cells observed in the graft tissue after 5 weeks showed expression of vimentin and keratocan, a corneal stromal marker (Fig. 5D, arrowheads). Interestingly, we observed Melan-A positive melanocytes in association with basal epithelial cells in the graft tissue similar to host tissue (Fig. 5D, arrowheads).

## Discussion

Decellularized corneal scaffolds recently gained interest as an alternative material for corneal replacement^26–28^. If corneal damage extends from the ocular surface to the upper stroma and an allotransplant is at high risk of rejection due to vascularization or inflammation, decellularized corneal scaffolds may serve as an alternative tissue source to reconstruct the corneal epithelium and its underlying stromal support. Therefore, it was our goal to devise an efficient, fast, and gentle method of decellularization using well-tolerated substances to generate human corneal scaffolds from readily available donor material.

Various protocols have been used to decellularize human corneal tissue, of them, SDS or hypertonic salt with nucleases were most effective in removing cellular components^6,7^. The combination of SD, a natural bile salt, and DNAse was used to decellularize human corneal limbus. However, significant drawbacks were the long elution times required for removal of cellular components by SDS/nucleases or NaCl/nucleases treatment reported for human corneal decellularization (~ 5–8 days)^6,7^ and SD/DNAse for treatment of human limbal tissue (~ 5 days)^18^. In this study, we established and validated a rapid alternative method (~ 1 day) for efficient decellularization of the human cornea using SD and DNAse. Our data indicate that 1% or 4% SD with DNAse and 4% dextran allow for efficient decellularization by removal of cellular components (HLA-ABC and nuclear debris) within 24hrs, in line with earlier reports on other tissues^11,17,18^.

Due to its high glycosaminoglycan content, corneal tissue has a strong tendency to swell in cell culture media^29–31^. A concentration of 5–6% dextran in a medium is therefore routinely used to maintain graft thickness prior to keratoplasty^31,32^. The significant difference of the mean NHC thickness between DM^−^ and DM^+^ groups is due to the presence or absence of dextran in the respective culture medium used for tissue transfer from the Cornea Bank to the laboratory. Most previous reports on human corneal decellularization have not specifically addressed tissue swelling^6,7^, but dextran use has been reported on porcine corneas in conjunction with ultrahigh hydrostatic pressure and chemical detergents^20,29,30^. We compared different concentrations of dextran to control tissue swelling during decellularization of the human cornea and found that the presence (DM^+^) or absence (DM^−^) of Descemet membrane had an additional influence on tissue thickness (Fig. 1B–D). In both, DM^+^ and DM^−^ cornea, a dextran concentration of 4% was sufficient to preserve corneal thickness during decellularization. In line with earlier reports on porcine tissue^7,20,29,30^, SD/DNAse-mediated removal of cellular components and DNA from human corneal tissue were unhampered by the presence of dextran (Fig. 1D,E). Moreover, our decellularization protocol is also applicable to DM^+^ corneas, which might be usable for full-thickness corneal transplantation in the future.

As optical properties are highly relevant for the selection of materials for corneal reconstruction, we next assessed transparency and optical transmittance of DHC. It has been reported that ionic detergents solubilize both cytoplasmic and nuclear cell membranes, damage collagen, and denature proteins by disrupting protein–protein interactions^33,34^. In a previous study, SDS/DNAse-treated corneas had the greatest loss of transparency with most ECM disruption as compared to corneas treated with non-ionic detergents^7^. Even though SD is an ionic detergent, ECM disruption, or reduction of the sGAGs content were not observed similar to previous studies on other tissues^35^. In our own experiments, the transparency of DHC decreased with decellularization in the absence of dextran and recovered after incubation in glycerol for 24 h (Fig. 2B). This suggests that the decrease in corneal transparency was a result of increased corneal hydration rather than permanent structural alteration^6,29^. However, it has also been reported that glycerol causes additional collagen disorganization^36^ and may nevertheless mask structural damage inflicted by the decellularization procedure^7^. Herein, we suggest to include dextran during decellularization of the human cornea to reduce tissue swelling, loss of transparency, and to maintain tissue integrity.

Ex vivo cultivated limbal epithelial cells have been used to reconstruct the corneal epithelium in limbal stem cell-deficient patients^37–40^. The long term clinical outcome of limbal epithelial cell grafts mainly depends on the maintenance of stem/progenitor cells, which is regulated by the niche microenvironment including its ECM components and mechanical properties in vivo^39,41–44^. Moreover, ECM molecules play a key role in cell–cell interactions, cell adhesion, proliferation, migration, and response^25,45^. Thus, the integrity and preservation of ECM components are essential for further clinical use of the decellularized tissues. The published decellularization protocols have not reported on ECM component preservation in much detail^6,7^. To address this issue, we extensively studied ECM components and their possible alteration. The corneal BM collagens type IV, important in the structure and function of BM, and type XVIII, having an anti-angiogenic activity^46,47^, were well preserved by our decellularization protocol. Heparin sulfate proteoglycans (e.g. perlecan and agrin) and glycoproteins (e.g. fibronectin), which were well preserved in this study, play important roles in epithelial cell migration and proliferation^48^. LNs are among the best described BM components of the cornea, of which the cornea-specific isoform LN-332 promotes adhesion, migration, and differentiation of LEPC^25,49^. Our own data clearly indicate the preservation of a vast panel of ECM proteins including various laminin isoforms as detected by immunofluorescence microscopy (Fig. 3).

Successful repopulation with cells is a prerequisite for the use of decellularized scaffolds in tissue engineering. It has been reported that structurally damaged decellularized cornea and residual decellularizing agents (SDS) detrimentally affect cell growth and proliferation^7^. It has been shown that SD-produced scaffolds were highly biocompatible compared to those decellularized using SDS^23^. We explored different strategies of DHC repopulation by plating cells or by allowing cellular invasion from host tissue. With both approaches, the repopulating epithelial cells maintained a proper phenotype as characterized by expression of the progenitor markers CK15, P-cadherin, and p63 in basal layers and the marker for differentiated epithelial cells, CK3, in the superficial layers. Stromal repopulation is a slower process as has been clinically observed in keratoconus patients following collagen cross-linking treatment. Activated keratocytes started to populate the corneal stroma at 2 months after treatment and repopulation was almost complete after 6 months^50^. We observed stromal repopulation of a scaffold by the 3rd week in vitro, whereas it took 5 weeks in an ex vivo model. Thus, the repopulation of DHC by stromal cells could find an application treating anterior corneal lesions where corneal stroma is altered by disease, injury, or scarring^51^. Currently, no data are available regarding the repopulation of decellularized tissue by LMs, which are found in close proximity to LEPC at the limbus in vivo, where melanocytes appear to play a supporting role in preserving the stemness state of corneal epithelial cells and modulating their migration towards the central cornea during regeneration^52,53^. LMs were able to adhere and intermingle with epithelial cells on DHC tissue, strongly suggesting the potential of the scaffold to facilitate LM survival. Moreover, in ex vivo lamellar grafting experiments we have also observed the migration of LMs from donor limbal tissue onto decellularized corneal grafts similar to reports on ocular surface regeneration in vivo^52,54^. All these observations indicate that the DHC scaffold provides a niche microenvironment to keep basal epithelial cells in a less differentiated state. Thus, it may serve as an ideal scaffold for future clinical transplantation purposes^6,45^. However, in vivo studies are necessary to determine whether the DHC supports stromal dehydration, long-term transparency, and in vivo biocompatibility. Moreover, it has to be tested whether a significant reduction of immunogenic epitopes was achieved to allow for long-term immunological tolerance.

In conclusion, we have developed a fast and efficient corneal decellularization method using clinically applicable SD with DNAse in the presence of dextran. Dextran prevented corneal swelling during decellularization. The biomechanical and optical properties, ECM architecture and BM composition of the DHC were well preserved and resembled the properties of cultured NHC. The DHC scaffolds revealed excellent biocompatibility and ex vivo transplanted corneas were completely epithelialized and effectively supported stromal cell ingrowth. Hence, this could be a promising scaffold for anterior corneal reconstruction in the treatment of corneal defects.

## Methods

### Tissue

Organ cultured human corneoscleral tissue remnants after DMEK (DM^−^, n = 102) and corneal buttons unsuitable for transplantation (DM^+^, n = 51) were used (Table 2). To isolate primary limbal cells for repopulation experiments, organ cultured corneoscleral rims remaining after penetrating keratoplasty were used (labeled after PK, n = 30, Table 2). The tissue was kindly provided by the LIONS Cornea Bank Baden-Württemberg, located at the Eye Center, University of Freiburg and informed consent for research use of remnant tissue had been given by the donors or their next of kin. The study was approved by the institutional review board of the University of Freiburg (25/20) and followed the tenets of the Declaration of Helsinki. No organs or tissues from prisoners were used.

Following the standard procedures for corneal grafts in the Eye Center, we obtained DM^+^ corneas from the Cornea Bank cultured (12.5 ± 8.4 days) in Carry-C medium (Alchimia) containing 6% dextran, whereas DM^−^ corneas were cultured also in Carry-C medium (1.4 ± 1.1 days) before DMEK surgery and were supplied to the laboratory in Tissue-C medium (Alchimia) without dextran. Due to higher availability, most experiments were performed on DM^−^ corneas except as otherwise indicated. DM^+^ corneas were especially used to comparatively analyze the decellularization protocols in the course of initial experiments.

### Decellularization

Corneas without any further processing were used as controls (NHC). For decellularization, all washing steps and SD (Sigma-Aldrich, Hamburg, Germany) incubation were carried out under continuous agitation (800 rpm) at room temperature. Corneoscleral tissue was washed in Dulbecco’s phosphate-buffered saline (DPBS; 3 × 5 min), placed in 12-well plates with 0.5%, 1%, or 4% SD in ultrapure water for 30 min, and then rinsed in DPBS (3 × 30 min). Subsequently, the tissues were incubated in DNAse I , 1 mg/ml in DPBS (Roche, Mannheim, Germany) overnight under a sterile hood and terminally washed in DPBS (4 × 30 min). The whole decellularization procedure was carried out under aseptic conditions to ensure tissue sterility.

### Quantification of tissue swelling

To assess the effects of dextran on corneal swelling during decellularization, all decellularization steps were performed in the presence of 0, 2, 4, or 6% dextran 500 (Sigma-Aldrich, Hamburg, Germany) in both DM^+^ and DM^−^ corneas. To verify the effect of dextran alone on NHC, DM^−^ corneas in incubated in 6% dextran (dextran control) and DM^+^ corneas in dextran free medium (dextran-free control) with an incubation time equivalent to the decellularization process. Corneal swelling was analyzed by AS-OCT using a clinical AS-OCT device (SS-1000 CASIA, Tomey, Nuernberg, Germany). Measurements of the central corneal thickness were performed with 3-dimensional cross-sectional imaging before and after decellularization as described earlier^55^.

### Histology

For bright field light microscopy, tissue was cryosectioned and stained as follows: After embedding and freezing in optimal cutting temperature medium, 10 µm thick sections were cut with a cryostat (Leica, Germany), mounted on adhesive slides and air-dried. Sections were stained with hematoxylin (Haematoxylin Gill III, Surgipath, Leica, Germany) for 2 min and 1% eosin Y (Surgipath, Leica, Germany) for 1 min to observe the gross tissue architecture and degree of decellularization. To visualize the proteoglycan content, cryosections were stained with 1% alcian blue (Morphisto, Germany) for 30 min together with a counterstain of nuclear fast red (Morhphisto, Germany). For glycoproteins, PAS staining was performed using 1% periodic acid (Fluka, Germany) for 10 min, and Schiff reagent (Roth, Germany) for 90 s. Samples were examined using either a bright field fluorescence microscope (Axio Imager.A1, Zeiss) and images were processed using ProgRes CapturePro Software (JENOPTIK, https://www.jenoptik.com/products/cameras-and-imaging-modules/microscope-cameras/progres-usb-20-firewire/software-download-progres) or a Hamamatsu NanoZoomer S60 (Hamamatsu Photonics, Herrsching, Germany).

### Immunostaining of frozen sections

Immunostaining on frozen sections performed as previously described^60^. Briefly, human corneas in optimal cutting temperature medium were cut into 10 µm sections, fixed in 4% paraformaldehyde (PFA) for 20 min or acetone for 10 min followed by permeabilization in 0.3% Triton X-100 in PBS for 10 min. The sections were blocked with 10% normal goat serum (NGS) and incubated in primary antibodies (Supplementary Table S1) diluted in 1% NGS in PBS overnight at 4 °C. Fluorescein isothiocyanate-conjugated or rhodamine-conjugated anti-mouse or -rabbit immunoglobulins (Life Technologies, Carlsbad, CA) were used for detection and nuclear staining was performed with DAPI (Vectashield antifade mounting medium with DAPI; Vector, Burlingame CA). Immunolabeled cryosections were examined with a laser scanning confocal microscope (TCS SP-8, Leica, Wetzlar, Germany). For negative controls, the primary antibodies were replaced by equimolar concentrations of an irrelevant isotypic primary antibody of the same species.

### Immunostaining of paraffin sections

Immunohistochemistry was performed as previously described^56^. The list of antibodies is provided in Supplementary Table S1.

### Scanning electron microscopy

SEM was performed as described earlier^57^. Briefly, scaffolds were fixed with 3.8% formaldehyde in PBS for at least 1 h and rinsed twice with PBS. Then, the scaffolds were dehydrated by rinsing through graded ethanol/water mixtures (50%, 70%, 80%, 90%, and 100%; each step for 10 min at room temperature). Next, ethanol was slowly exchanged by liquid CO_2_ (critical point dryer; Balzers CPD, Balter's Union) and the samples were dried using the critical point method. For cross-sections, the specimens were sectioned transversely. Finally, whole tissue and sections were sputtered with a thin layer of gold of ∼10 nm in thickness (sputter coater, Balzers SLD; Balzers Union), and documented by a Leo 435VP (Zeiss) scanning electron microscope.

### Elastic modulus determination by instrumented indentation

Biomechanical characterization was performed as published earlier^58^. In brief, the tissue was immersed in a medium containing 15% dextran for 24 h to achieve dehydration similar to the situation in vivo. Central tissue samples were cut with a 7 mm diameter trephine and the tissue was glued to the bottom of a cell culture dish. The indentation was performed using a spherical ruby tip of a 500 µm radius on a Bioindenter (Anton Paar, Peseux, Switzerland). Load-controlled measurements with loading and unloading rates of 300 µN/min and a maximum indentation load of 200 µN were taken. The elastic modulus was calculated using the Hertz model and Bioindenter indentation software (Anton Paar, https://wiki.anton-paar.com/en/indentation-testing-biological-soft-materials-using-bioindenter/).

### DNA content

DNA was extracted using the DNeasy Blood & Tissue Kit (69,504, Qiagen, Hilden, Germany). The central 6 mm diameter of NHC and DHC were trephined and incubated with proteinase K in ATL buffer at 56 °C under continuous agitation and further processed for DNA elution according to the manufacturer’s protocol. The eluates were analyzed photometrically at 260 nm wavelength using the NanoDrop OneC Microvolume UV–Vis spectrophotometer (Thermo Scientific).

### Optical properties

The optical properties of the NHC and DHC were assessed by a UV–Vis spectrophotometer as described previously^59^. Breifly, the 6 mm diameter central corneal pieces were trephined from both NHC and DHC and the corneal pieces were placed in a 96-well UV-star microplate (Greiner, Germany) filled with glycerol. The spectral transmittance of each sample was measured using a spark microplate reader (TECAN) and the absorbance data were recorded at 1 nm wavelength increments (300–750 nm). The transmittance of the samples was corrected with glycerol as a blank medium and data were analyzed as the mean percentage of transmittance. In addition, photographs (Digital camera, Canon EOS 700D) were taken with the word “cornea” behind the corneal tissue to give a visual representation of the differences in transparency.

### Sulfated glycosaminoglycans

The sGAG content of DHC was determined using a 1,9-dimethyl methylene blue (DMMB assay) (280,560-N, Proteoglycan detection kit, Amsbio) according to the manufacturer’s protocol. The central 6 mm of the corneas were trephined and digested with papain as per manufacturer’s protocol (Tissue digestion kit, Amsbio). Briefly, the tissue was homogenized, and digested with papain at 60 °C for 1 h, and acetic acid and Tris-hydrochloric acid were added. The digested samples were added to DMMB and absorbance was measured at 515 nm using a spark microplate reader (TECAN). The absorbance was expressed in percentage (%) with reference to NHC (100%).

### Cell culture

LEPC, LMSC, and LM were isolated, cultivated, and characterized as described earlier^60^, details are provided in the Supplementary Data S1.

### Repopulation of decellularized corneas with cultured cells

For recellularization and ex vivo transplantation experiments 4% dextran and 1% SD were used during the decellularization process. For DHC-LMSC scaffolds, the isolated LMSC (P2) were injected (20 µl) at 10 locations in the decellularized corneal stroma (about 20% depth from the posterior side) with a 20-gauge needle on a 1 ml syringe (1 × 10^6^ cells/ml). The corneas with injected cell suspensions were transferred to 12-well plates containing Mesencult media (Stem Cell Technologies). For DHC-LEPC scaffolds, the isolated human LEPC (P1) were seeded (1 × 10^6^ cells/ml) on the anterior surface of the cornea and cultured in corneal culture medium (CCM) containing DMEM/ Ham’s F12 (3:1) (Hyclone; GE Healthcare Life Sciences, Freiburg, Germany) supplemented with human corneal growth supplement (Life Technologies), 5% FCS (GE Healthcare Life Sciences) and low calcium concentration (0.4 mM Ca^2+^, labeled CCM-low). For DHC-LEPC/LMSC scaffolds, LMSCs were injected in DHC as mentioned earlier, followed by LEPCs seeding 24 h later and cultivation of the resulting constructs in CCM-low. For DHC-LEPC/LM scaffolds, both LEPC and LM were seeded together in a ratio of 3:1 on the decellularized corneal surface and cultured in CCM-low medium. For stratification of DHC-LEPC, -LEPC/LMSC, and -LEPC/LM scaffolds, after 1 week, the tissues were raised to the air–liquid interface and the CCM was shifted to high-calcium concentrations (2 mM Ca^2+^, labeled CCM-high) and cultured further 2 weeks. All cultures were maintained at 37 °C, 5% CO_2_, and 95% humidity and medium was changed every alternative day. During culturing, recellularized scaffolds were examined using phase contrast microscope (Axio Vert.A1, Zeiss) and images were processed using ProgRes CapturePro software (JENOPTIK, https://www.jenoptik.com/products/cameras-and-imaging-modules/microscope-cameras/progres-usb-20-firewire/software-download-progres). After terminating the cultivation, corneal samples were fixed for immunohistochemistry and light microscopy as described above.

### Cell viability

Live/dead viability/cytotoxicity kit (MP 03,224, Molecular Probes) was used to visualize live and dead cells in repopulated DHC as per manufacturer's protocol. Briefly, the repopulated DHC was incubated for 30 min at room temperature in calcein-AM (2 µM) and ethidium homodimer (4 µM) solution. After incubation, the cornea was rinsed thrice in PBS and images were photographed using a inverted fluorescence microscope with ZEN software (Axio Observer Z1, Zeiss; https://www.zeiss.com/microscopy/int/products/microscope-software/zen.html).

### Sutureless repopulation of decellularized corneas

In order to mimic the in-vivo-system in a sutureless setup to avoid damage to the ECM, examined the gradual repopulation of the DHC by mounting a native corneoscleral tissue quarter in close contact to a decellularized scaffold quarter. Details of the experiment are provided in the Supplementary Data S2.

### Ex vivo transplantation

The feasibility of the DHC to serve as donor tissue for corneal transplantation was examined by performing anterior lamellar keratoplasty (ALK) ex vivo on non-decellularized human cadaveric corneoscleral tissue. For ALK, a microkeratome (mk-DSAEK, Gebauer, Neuhausen, Germany) was used to dissect both the NHC (DM^+^) and the DHC (DM^−^) tissue using a 450 µm keratome head. Using a surgical microscope (OPMI VISU 140, Zeiss, Oberkochen, Germany) and an artificial anterior chamber (Barron Artificial Anterior Chamber, Katena Products, INC., Parsippany, USA) to fix the host tissue, the anterior corneal part of the DHC tissue (graft) was placed epithelial side up on the posterior bed of the NHC (host). These constructs were fixed temporarily with four opposing single stitches 90 degrees apart from each other using polyamide suture material (10–0 Ethilon, ETHICON, Johnson & Johnson Medical Devices, Ohio, USA). Subsequently, the tissues were definitely attached with double running cross-stitch sutures^61^. Finally, the four temporary sutures were removed. Thereafter, the corneal samples were transferred into 12-well plates and cultured in CCM-high medium for 5 weeks.

### Statistics

Statistical analyses were performed using GraphPad Prism software (Version 6.0; Graphpad Software Inc., La Jolla, CA; https://www.graphpad.com/). Data is represented as a mean ± standard deviation (S.D.) from individual experiments (Table 1 & 2) or as mean ± standard error of the mean (S.E.M.) (graphs). The statistical significance (*p* ≤ 0.05) was evaluated by the Wilcoxon signed-rank test or Mann–Whitney U test.

## Supplementary Information

Supplementary Information

## Data Availability

The datasets generated during and /or analyzed during the current study are available from the corresponding author on reasonable request.
