# Supplementary material for: A decellularized human corneal scaffold for anterior corneal surface reconstruction
Source: Sci Rep. 2021 Feb 4;11:2992. doi: 10.1038/s41598-021-82678-3 (PMC7862698; doi:10.1038/s41598-021-82678-3)
Supplement: Supplementary file 1 — Supplementary Information [file 41598_2021_82678_MOESM1_ESM.pdf]

A decellularized human corneal scaffold for anterior corneal surface reconstruction

Naresh Polisetti[1], Anke Schmid[1], Ursula Schlötzer-Schrehardt[2], Philip Maier[1], Stefan Lang[1], Thorsten Steinberg[3], Günther Schlunck[1], Thomas Reinhard[1]

[1]Eye Center, Medical Center - Faculty of Medicine, University of Freiburg, Killianstrasse 5, 79106, Freiburg, Germany

[2]Department of Ophthalmology, University Hospital Erlangen, Friedrich-Alexander-University of Erlangen-Nürnberg, Schwabachanlage 6, D-91054 Erlangen, Germany

[3]Department of Operative Dentistry and Periodontology, Division of Oral Biotechnology, University of Freiburg, Hugstetter Strasse 55, 79106, Freiburg, Germany.

Supplementary Data 1

Cell culture and characterization: Limbal cell isolation was carried out as described previously from organ cultured corneoscleral rims remaining after penetrating keratoplasty[59]. Briefly, epithelial-mesenchymal-melanocyte clusters were generated by digestion of limbal tissue with collagenase A (Sigma). Isolated limbal clusters were subjected to trypsinization to get a single-cell suspension. Single-cell suspensions were seeded into T75 flasks (Corning, Tewksbury, MA) in keratinocyte serum-free medium (KSFM) supplemented with bovine pituitary extract, epidermal growth factor (Life Technologies) and 1x penicillin-streptomycin mix (Pan Biotech) to enrich limbal epithelial progenitor cells (LEPC); For limbal mesenchymal stromal cells (LMSC), single-cell suspensions were seeded into T75 flasks in Mesencult media (Stem Cell Technologies); For limbal melanocytes (LM), limbal cultures treated with geneticin and enriched melanocytes cultured in CNT-40 medium (Cellntec). All cultures were incubated at 37 ^{0}C under 5 % CO_2 and 95 % humidity, with a medium change every second day. The cells were characterized by quantitative real-time polymerase chain reaction, flow cytometry, and immunofluorescence as described[25,59]. The list of all primers is available in Supplementary Table S2.

Results: The cultured LEPC, LMSC, and LM were successfully established (Supplementary Fig. 1A). The gene expression analysis showed LEPC cells expressing the progenitor markers cytokeratin 15 (KRT15 or CK15), P-cadherin (CDH3) (Supplementary Fig. B). The cell-cell adhesion molecule E-cadherin (CDH1) and cell-matrix adhesion molecule integrin α3 (ITGA3) were also highly expressed in LEPC (Supplementary Fig. B). Moderate expression of the proliferation marker Ki-67 was observed, whereas no expression of the corneal differentiation marker cytokeratin 3 (KRT3) was observed (Supplementary Fig. B). Flow cytometer analysis of LMSCs revealed that all cells expressed the mesenchymal markers CD44, CD73, and CD90 and were negative for CD11c, CD14, CD19, and CD45 confirming their mesenchymal phenotype (Supplementary Fig. C). LMs expressed Melan-A, human melanoma black-45, tyrosinase-related protein 1, and sex-related HMG box 10, which are characteristic markers for LM (Supplementary Fig. D).

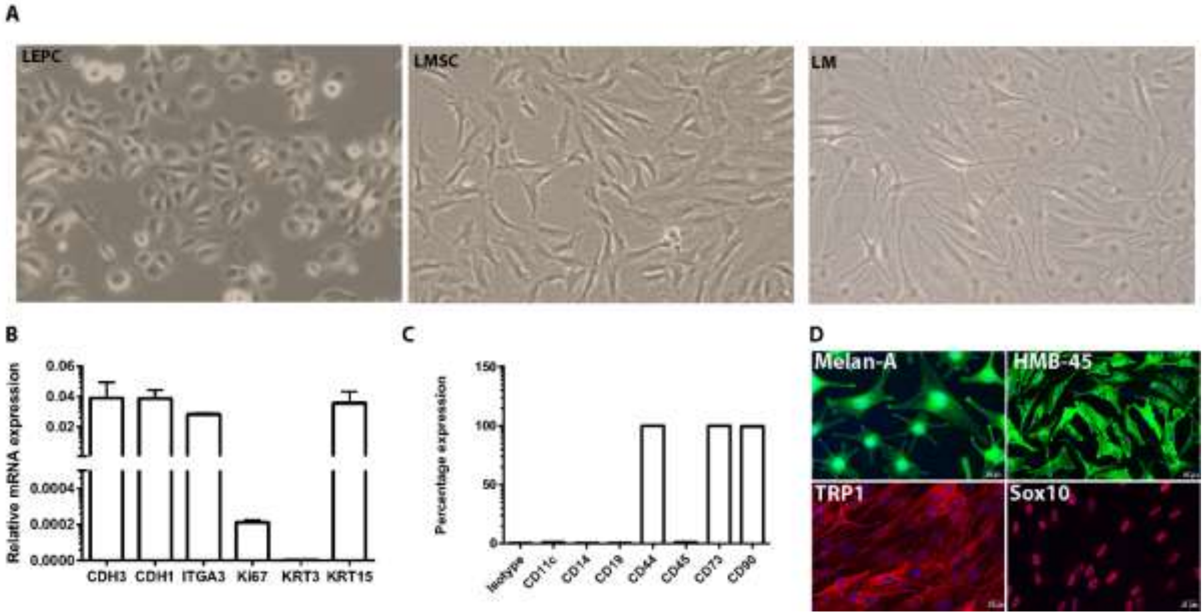

Supplementary Figure S1: Culturing and characterization of Limbal cell cultures: A) Phase-contrast micrographs of cultured limbal epithelial progenitor cells (LEPC), limbal mesenchymal stromal cells (LMSC), and limbal melanocytes (LM). Magnification x100. B) Gene expression profile of LEPC by a quantitative real-time polymerase chain reaction. Graphs represent the relative mRNA expression of genes on cultured LEPC cells. C) Flow cytometry analyses of cultured LMSC showing positive expression of CD44, CD73, and CD90, but a negative expression of CD11c, CD14, CD19, and CD45. Percentages (%) of positive cells are expressed as means ± S.E.M. (n=3). D) Immunostaining cultured LMs expressing Melan-A, HMB-45, TRP1, and SOX10. Nuclei counterstained with DAPI. Abbreviations: CDH3, P-cadherin; CDH1, E-cadherin; ITGA3, integrin alpha 3; KRT15 or

CK15, cytokeratin 15; KRT3, cytokeratin 3; SOX10, sex-related HMG box 10; TYRP1 or TRP1, tyrosinase-related protein 1; HMB45, human melanoma black-45; DAPI, 4',6-diamidino-2-phenylindole.

Supplementary Data 2

Sutureless repopulation of decellularized corneas: In order to mimic the *in-vivo*-system in a sutureless setup to avoid damage to the ECM, we examined the gradual repopulation of the DHC by native neighboring tissue. Each corneoscleral button was divided into quarters of which two quarters underwent decellularization process as described in the main article (DHC) and two quarters remained untreated as NHC. Subsequently, two corneoscleral quarters, one NHC and one DHC were attached next to each other using a warm, liquid agarose gel (1.5 % gel; Agarose, Sigma). In doing so, we aimed at an optimal tissue adaptation avoiding gap formation between the two corneal parts as good as possible. The agarose was then allowed to cool down for some minutes before adding the CCM-high media followed by incubation at 37 ^{o}C, 5 % CO_2, and 95 % humidity for up to 5 weeks. Immunohistochemistry of samples processed as mentioned in the main article file.

Results: The sutureless NHC and DHC cornea attached next to each other in close contact on an agarose gel as depicted in Supplementary. Fig. 2A. After one week, histological analyses showed the coverage of DHC by NHC epithelium (Supplementary. Fig. 2B) and stratification of the epithelium was noted in samples after three weeks of cultivation (Supplementary Fig. 2C). The stromal repopulation of DHC by NHC cells was noted after two weeks and maximized after 5 weeks of cultivation, as confirmed by PAS staining (Supplementary Fig. 2C). Immunfluorescence staining analysis revealed the expression of pan-CK in DHC and stromal cell migration (vimentin expression) observed in 2nd week and complete coverage by 5th week (Supplementary Fig. 2D). Immunofluorescence analysis showed expression of cytokeratin 3 (CK3) on superficial cells and p63 expression on basal cells on 2nd and 5th-week samples (Supplementary Fig. 2E).

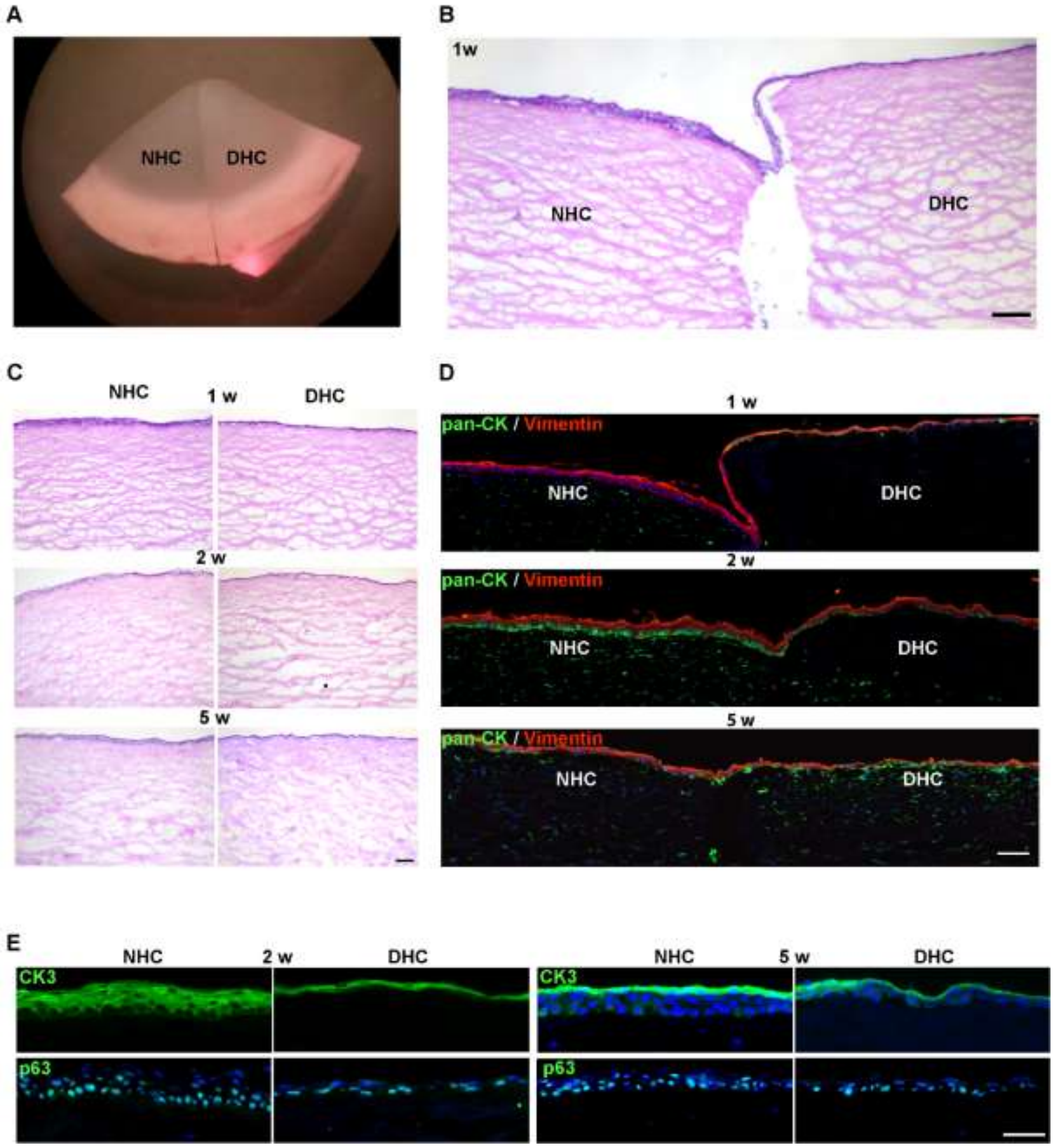

Supplementary Figure S2: Sutureless transplantation: A) The sutureless NHC and DHC corneal quarters were attached next to each other in close contact on agarose gel. B & C) After one week, PAS staining showing the coverage of DHC by NHC epithelium and stratification of the epithelium in samples after 3 and 5 weeks of cultivation. The stromal repopulation of DHC was noted after two weeks and maximized after 5 weeks of cultivation. D) Immunofluorescence staining analysis showing the expression of cytokeratin (pan-CK) in all cells on the epithelial part of the DHC and commencing stromal cell migration (vimentin expression) in 2nd week, completed by 5th week. E) Immunofluorescence analysis showing expression of cytokeratin 3 (CK3) on superficial cells and p63 expression on basal cells on 2nd and 5th-week samples.

Supplementary tables

Supplementary Table S1: List of antibodies used

**Antibody (clone), Host species**	**Antibody dilution**	**Application**	**Antibody source**
Agrin, rabbit	1:2000	Immunohistochemistry	R. Timpl / T. Sasaki
Cadherin-E (24E10), rabbit	1:200	Immunohistochemistry	Cell signaling
Cadherin-P (A-10), Mouse	1:100	Immunohistochemistry	Santa Cruz Biotechnology, INC.
CD11c, PE(3.9), Mouse	5 µl/10^6 cells	Flow cytometry	BioLegend
CD14 PE(M5E2), Mouse	5 µl/10^6 cells	Flow cytometry	BioLegend
CD19 PE(H1B19), Mouse	5 µl/10^6 cells	Flow cytometry	BioLegend
CD44 PE(IM7), Mouse	5 µl/10^6 cells	Flow cytometry	BioLegend
CD45 PE(H130), Mouse	5 µl/10^6 cells	Flow cytometry	BioLegend
CD73 PE(AD2), Mouse	5 µl/10^6 cells	Flow cytometry	BioLegend
CD90 PE(5E10), Mouse	5 µl/10^6 cells	Flow cytometry	BioLegend
Collagen III, rabbit	1:100	Immunohistochemistry	Abcam
Collagen IV (2F11), mouse	1:100	Immunohistochemistry	Southern Biotech
Collagen XVIII, rabbit	1:2000	Immunohistochemistry	R. Timpl / T. Sasaki
Cytokeratin pan (PCK-26), mouse	1:500	Immunohistochemistry	Abcam
Cytokeratin 3 (AE5), mouse	1:100	Immunohistochemistry	Abcam
Cytokeratin 15 (LHK15), mouse	1:500	Immunohistochemistry	Abcam
Cytokeratin 15 (EPR1614Y), rabbit	1:500	Immunohistochemistry	Abcam
IgG2a, k, isotype PE (MOPC-173), Mouse	5 µl/10^6 cells	Flow cytometry	Biolegend
HMB-45 (HMB45), mouse	0.5 µg/ml	Immunocytochemistry	Abcam
Fibronectin (IST-4), mouse	1:100	Immunohistochemistry	Sigma-Aldrich
JAM-C (CRAM-19H36), rat	1:100	Immunohistochemistry	Serotec (Biorad)
Keratocan (aa196-245), rabbit	1:100	Immunohistochemistry	LSBio
Ki-67 (EPR3610), rabbit	1:500	Immunohistochemistry	Abcam
Laminin α3, rabbit	1:1000	Immunohistochemistry	R. Timpl / T. Sasaki
Laminin α5 (504), rabbit	1:1000	Immunohistochemistry	L. Sorokin, Münster
Laminin β2 (409), rabbit	1:1000	Immunohistochemistry	L. Sorokin, Münster
Laminin β3, rabbit	1:1000	Immunohistochemistry	R. Timpl / T. Sasaki
Laminin γ2, rabbit	1:1000	Immunohistochemistry	R. Timpl / T. Sasaki
Melan A, (EPR20380), rabbit	1:1000	Immunohisto/cytochemistry	Abcam
p63 (Y289), rabbit	1:200	Immunohistochemistry	Abcam
Perlecan	1:500	Immunohistochemistry	R. Timpl / T. Sasaki
Sox10 (EPR4007), rabbit	1:250	Immunocytochemistry	Abcam
Tenascin C, rabbit	1:200	Immunohistochemistry	Millipore
TRP1 (EPR21960), rabbit	1:1000	Immunocytochemistry	Abcam
Vimentin, (D21H3), rabbit	1:500	Immunohistochemistry	Cell Signaling
Vitronectin (8E6), mouse	1:100	Immunohistochemistry	Millipore

Supplementary Table S2: Primers used in qRT-PCR primer assays

Gene Symbol	Accession No.	Product Length (nt)	Probe No. /SYBR	Sequence 5 ‘ – 3’
CDH1	NM_001317186.1	72	P35	CCCGGGACAACGTTTATTAC
				GCTGGCTCAAGTCAAAGTCC
CDH3	NM_001793.5	83	P15	AGGGAGGCTGAAGTGACCTT
				GGGCAGCCCATGAATACTT
GAPDH	NM_002046.5	120	P45	GAGTCCACTGGCGTCTTCAC
				GTTCACACCCATGACGAACA
Ki67	NM_002417.4	78	P39	CCAACCAAAAGAAAGTCTCTGG
				TGATGGTTGAGGCTGTTCCT
ITGA3	NM_002204.3	75	P13	GAGGACATGTGGCTTGGAGT
				GTAGCGGTGGGCACAGAC
KRT3	NM_057088.2	87	P31	TGAGCTGAAGAACATGGAGGA
				TCATTCTCAGCAGCTGTACGTT
KRT15	NM_002275.3	104	P77	TACTTACCGCAGCCTGCTC
				TGATGTGGAAATTGCTGCTG

Primers were used for Probe based (Universal Probe Library) qRT-PCR assays with an annealing temperature of 60°C.
